# Response to commentaries: (de)normalization of drinking and its implications for young people, sociality, culture and epidemiology

**DOI:** 10.1111/add.15848

**Published:** 2022-02-28

**Authors:** Gabriel Caluzzi, Michael Livingston, John Holmes, Sarah MacLean, Dan I. Lubman, Paul Dietze, Rakhi Vashishtha, Rachel Herring, Amy Pennay

**Affiliations:** ^1^ Centre for Alcohol Policy Research La Trobe University Bundoora Australia; ^2^ National Drug Research Institute Curtin University Perth Australia; ^3^ School of Health and Related Research University of Sheffield Sheffield UK; ^4^ School of Allied Health, Human Services and Sport La Trobe University Bundoora Australia; ^5^ Turning Point, Eastern Health Melbourne Australia; ^6^ Monash Addiction Research Centre, Eastern Health Clinical School Monash University Melbourne Australia; ^7^ National Drug Research Institute and EnAble Institute Curtin University Perth Australia; ^8^ Behaviours and Health Risks Program Burnet Institute Melbourne Australia; ^9^ Drug and Alcohol Research Centre Middlesex University London UK

**Keywords:** Alcohol, alcohol epidemiology, cultural change, declining youth drinking, digital technology, normalization, sociality

*Processes of (de)normalization of drinking are likely to be underpinned by broader changes in young people's lives. It is important to continue examining what this means from a social, cultural and epidemiological perspective*.

Our recent article [1] has prompted important questions from cultural studies [2], sociological [3] and epidemiological [4] perspectives. Alasuutari [2] is critical of the ability of the normalization thesis to account for changes in young people's drinking, suggesting that it provides a description of change rather than an explanation. Alasuutari further emphasizes the importance of social media and digital technologies as broad transnational drivers. Our article explored the question: ‘has non‐drinking become normalized, and/or has drinking become de‐normalized, for young people?’. We did not, however, explore the reasons behind these social processes occurring. We agree wholeheartedly that processes of normalization must be understood in the context of broader changes in adolescents’ lives. This fits with the way normalization was first theorized—as inextricably tied up with broader social and economic changes occurring for young people in the mid‐1990s [5]. Indeed, a substantial body of scholarly work has explored these shifts in relation to economic precarity, concerns about health and wellness, policy changes, evolving values and attitudes to alcohol, changes in digital technology use and family relationships [6, 7, 8, 9, 10, 11, 12, 13]. These factors—common to high‐income countries—provide the broader context underpinning our argument.

While the rise of digital technologies has fundamentally changed socializing, overemphasizing digital technologies as a driver for declining trends risks a return to individual drivers, rather than ‘big picture’ theories entailing complex social changes. The effects of digital technologies are multi‐faceted, transforming young people's relationships with alcohol in complex ways [14]. A big picture explanation should seek to explore how technologies assemble, and are assembled by, other social and economic structures to shape young people's lives. Indeed, processes of (de)normalization are circular in that they both frame alcohol, but are also influenced by external forces such as technology.

Herold & Kolind [3] are—perhaps rightly—unconvinced that non‐drinking has become normalized for young people in Denmark. They point to literature showing that drinking is still prevalent and remains an important facilitator of sociality, gender performance and a marker of adulthood. However, most of the literature they draw upon comes from young adults, rather than adolescents (who are the subjects of our argument), and it would be interesting to know how these processes play out for younger Danes. We agree that it is important to include research with heavy drinkers as a necessary component in understanding large shifts in drinking for young people, especially concerning the importance of drinking contexts [1]. Further, the continued prevalence and importance of alcohol for young Danes (to a much greater degree than their Scandinavian neighbours [15]) highlights the value of cross‐national comparative research [16].

Finally, Rossow [4] asks an important question from an epidemiological point of view: as non‐drinking becomes normalized, will abstainers become less distinctive? Or we add as a counter‐hypothesis, as abstinence becomes less risky, will heavy drinking become riskier? Early studies from Sweden provide some evidence that recent adolescent abstainers are indistinguishable from drinkers on key socio‐demographic measures [17], a shift from earlier findings [18]. However, while the declines in adolescent drinking for recent cohorts are marked, these cohort differences tend to narrow with age [19], meaning abstention in adulthood has not increased dramatically as yet. Nevertheless, as Rossow reminds us, alcohol's shifting cultural position requires regular re‐assessments of long‐standing tenets of alcohol epidemiology.

## DECLARATION OF INTERESTS

None.

## AUTHOR CONTRIBUTIONS

**Gabriel Caluzzi:** writing ‐ original draft (lead); writing ‐ review and editing (equal). **Michael Livingston:** writing ‐ original draft (supporting); writing ‐ review and editing (equal). **John Holmes:** writing ‐ review and editing (equal). **Sarah MacLean:** writing ‐ original draft (supporting); writing ‐ review and editing (equal). **Dan Lubman:** writing ‐ review and editing (equal). **Paul Dietze:** writing ‐ review and editing (equal). **Rakhi Vashishtha:** writing ‐ review and editing (equal). **Rachel Herring:** writing ‐ review and editing (equal). **Amy Pennay:** writing ‐ original draft (supporting); writing ‐ review and editing (equal).
